# Distribution of legal retail cannabis stores in Canada by neighbourhood deprivation

**DOI:** 10.1186/s42238-023-00211-x

**Published:** 2024-02-14

**Authors:** Fathima Fataar, Pete Driezen, Akwasi Owusu-Bempah, David Hammond

**Affiliations:** 1 School of Public Health Sciences, University of Waterloo, 200 University Ave W, Waterloo, ON N2L 3G1 Canada; 2 Department of Psychology, University of Waterloo, Waterloo, ON Canada; 3 Department of Sociology, University of Toronto, Toronto, ON Canada

**Keywords:** Cannabis, Marijuana, Canada, Retail availability, Neighbourhood deprivation

## Abstract

**Objectives:**

In legal cannabis markets, the distribution of retail stores has the potential to influence transitions from illegal to legal sources as well as consumer patterns of use. The current study examined the distribution of legal cannabis stores in Canada according to level of neighbourhood deprivation.

**Methods:**

Postal code data for all legal cannabis stores in Canada were collected from government websites from October 2018 to September 2021. This data was linked to the Institut National de Santé Publique du Québec measures for material and social neighbourhood deprivation. Descriptive data are reported, including differences across provinces with different retail systems.

**Results:**

At the national level, there were approximately 8.0 retail cannabis stores per 100,000 individuals age 15+ in September 2021. The distribution of stores was closely aligned with the expected distribution across levels of material deprivation: for example, 19.5% of stores were located in neighbourhoods with the lowest level of material deprivation versus 19.1% in the highest level. More cannabis stores were located in the ‘most socially deprived’ or ‘socially deprived’ neighbourhoods (37.2% and 22.1%, respectively), characterized by a higher proportion of residents who live alone, are unmarried, or in single-parent families. The distribution of stores in provinces and territories were generally consistent with national patterns with a few exceptions.

**Conclusion:**

In the first 3 years following cannabis legalization in Canada, retail cannabis stores were evenly distributed across materially deprived neighbourhoods but were more common in socially deprived neighbourhoods. Future monitoring of retail store locations is required as the legal retail market evolves in Canada.

## Background

Canada legalized non-medical (‘recreational’) cannabis in October 2018 at the federal level. While the number of brick and mortar stores was initially limited, there has been substantial growth to more than 2400 stores by September 2021 (Canadian Centre on Substance Use and Addiction 2022). Prior to the legalization of non-medical cannabis, medical cannabis was available to authorized users through home grow or mail order from a licensed producer who was regulated by Health Canada (Shim et al. 2023). No ‘brick-and-mortar’ stores were permitted although some Canadian cities had unauthorized stores self-identifying as ‘medical dispensaries’ (Mahamad and Hammond 2018). Although legalization occurred at the federal level, provinces and territories are responsible for retail distribution of cannabis, including licensing cannabis retailers and establishing guidelines. Six provinces and all three territories have opted for privately run brick-and-mortar stores (Newfoundland and Labrador, Ontario, Manitoba, Saskatchewan, Alberta, Nunavut, Yukon, and Northwest territories), four provinces have opted for government-run stores (Prince Edward Island, Nova Scotia, New Brunswick, and Quebec), while British Columbia has a hybrid model. Although retail sales were permitted on the same date as legalization, the opening legal retail stores varied considerably between provinces following legalization. In some provinces such as Alberta and Manitoba, some legal ‘brick and mortar’ stores opened on the first day of legalization, while in other provinces, such as Ontario, the first stores opened months later (Wadsworth et al. 2023). Online sales of cannabis are legal in all jurisdictions in Canada and were available from the date of legalization Regulations surrounding the location of stores, such as distance from schools and distance from other cannabis stores, vary widely across provinces. In some jurisdictions, such as Ontario, a provincial regulation of 150 m distance from a school boundary line is required for all stores, with no requirements for distances between stores (Gagnon et al. 2022). In other cases, municipalities are allowed to set their own regulations on these matters (Gagnon et al. 2022).

The number and location of retail cannabis stores has the potential to influence patterns of use and purchasing behavior. This has been demonstrated for alcohol (Gmel et al. 2016) and tobacco (Cantrell et al. 2016; Reitzel et al. 2011) and more recently for cannabis (Wadsworth et al. 2021; Pedersen et al. 2021; Everson et al. 2019). With the changing landscape of legal cannabis retail stores, there is interest in understanding the distribution of stores based on neighbourhood deprivation. If stores are disproportionality located in deprived neighbourhoods, marginalized communities may be exposed to greater promotion of and access to cannabis, leading to greater use (Mair et al. 2015; Rhew et al. 2022). A recent study found that in Washington state, where non-medical cannabis is legally available, there was not only greater cannabis retail availability in disadvantaged neighbourhoods, but cannabis use and perceived acceptability of use were also higher (Rhew et al. 2022). Greater retail access to legal cannabis has been associated with current and frequent cannabis use (Everson et al. 2019; Shih et al. 2019; Ambrose et al. 2021). In addition, the availability of retail stores also has the potential to shape social norms within communities, which may impact subsequent use (Rhew et al. 2022). Conversely, a lack of legal stores in more deprived neighbourhoods could suppress transitions to the legal market and increase the risk of criminal sanctions from illicit cannabis. This is a particular concern given that individuals living in marginalized communities and racial minorities have been disproportionately targeted by criminal sanctions for cannabis possession (Wortley and Jung 2020; Owusu-Bempah and Luscombe 2020).

Research has examined the distribution of both medical dispensaries and retail stores in some US states by neighbourhood deprivation. For example, a 2009 study in California found that dispensaries were more likely to be found in areas with high cannabis demand, higher rates of poverty, and more alcohol outlets (Morrison et al. 2014), while a 2020 study found that there were more unlicensed outlets in low-income areas and more licensed outlets in areas where the majority of residents were white and had higher levels of education (Firth et al. 2022). In Colorado, more licensed retail outlets for medical and recreational cannabis were found in low income areas with a higher proportion of ethnic and racial minority groups (Shi et al. 2016). Similarly, a study in Washington State found that between 2014 and 2017, the density of recreational retail cannabis outlets was greatest in the most deprived neighbourhoods at all time points, with significantly more outlets in the most deprived neighbourhoods compared to the least deprived neighbourhoods (Amiri et al. 2019). Some have suggested that these communities lack social and economic resources to resist establishment of outlets in their neighbourhoods (Shi et al. 2016; Morrison et al. 2014). While there is little research on the distribution of retail stores in Canada since legalization in October 2018, one study found that, as of October 2020, there were almost 1.9 times the number of legal retail cannabis stores within 1000 m of the lowest income neighbourhoods compared to the highest income neighbourhoods, which was down from 2.4 times in October 2019 (Myran et al. 2019). However, the retail market has more than doubled since this period, and it is unclear if this trend will continue.

The current study sought to examine the distribution of physical legal retail cannabis stores in Canada overall and within each province/territory by neighbourhood deprivation using a comprehensive measure which considers not only income, but also other aspects of material and social deprivation.

## Methods

### Legal retail sources with storefronts

Official provincial and territorial government websites were used to identify a complete list of legal cannabis stores with storefronts in Canada beginning in November and December 2018 (shortly after legalization) up to September 7, 2021 (*n* = 2477). Websites were reviewed annually, and the list of stores were updated accordingly, including the removal of stores that were no longer listed. The postal code for each store location was recorded.

#### Neighbourhood deprivation index

All store postal codes were linked to a validated national database of neighbourhood deprivation indices from the Institut National de Santé Publique du Québec (INSPQ) (Gamache et al. 2019; Pampalon et al. 2012). The 2016 index is based on Canadian Census dissemination areas (DA), which served as a proxy for neighbourhoods. The DA is the smallest geographical unit of the census for which estimates are released and include 400–700 people per DA. Where data were available, each postal code in the country was assigned two scores: (1) a material deprivation score (based on the level of education, income, and employment in the population 15 and over) and (2) a social deprivation score (based on the proportion of the population aged 15 and over living alone, who are separated, divorced or widowed as well as the proportion of single-parent families). Each index is represented by quintiles on a scale of 1–5, with each group representing 20% of the dissemination areas (most privileged/privileged/neither deprived nor privileged/deprived/most deprived).

### Research design and analysis

A descriptive study design was used given that ‘census’ data on all stores was available without the need for any sampling; descriptive statistics are reported using SAS version 9.4. The proportion of stores within each level of material and social neighbourhood deprivation was estimated for Canada overall and by province/territory. Scores for each deprivation index were based on regional data (BC, Prairie provinces, Ontario, Quebec, Atlantic provinces) (Gamache et al. 2019). Where regional scores were not available, national deprivation scores were imputed (*n* = 58).

## Results

**Fig. 1:**
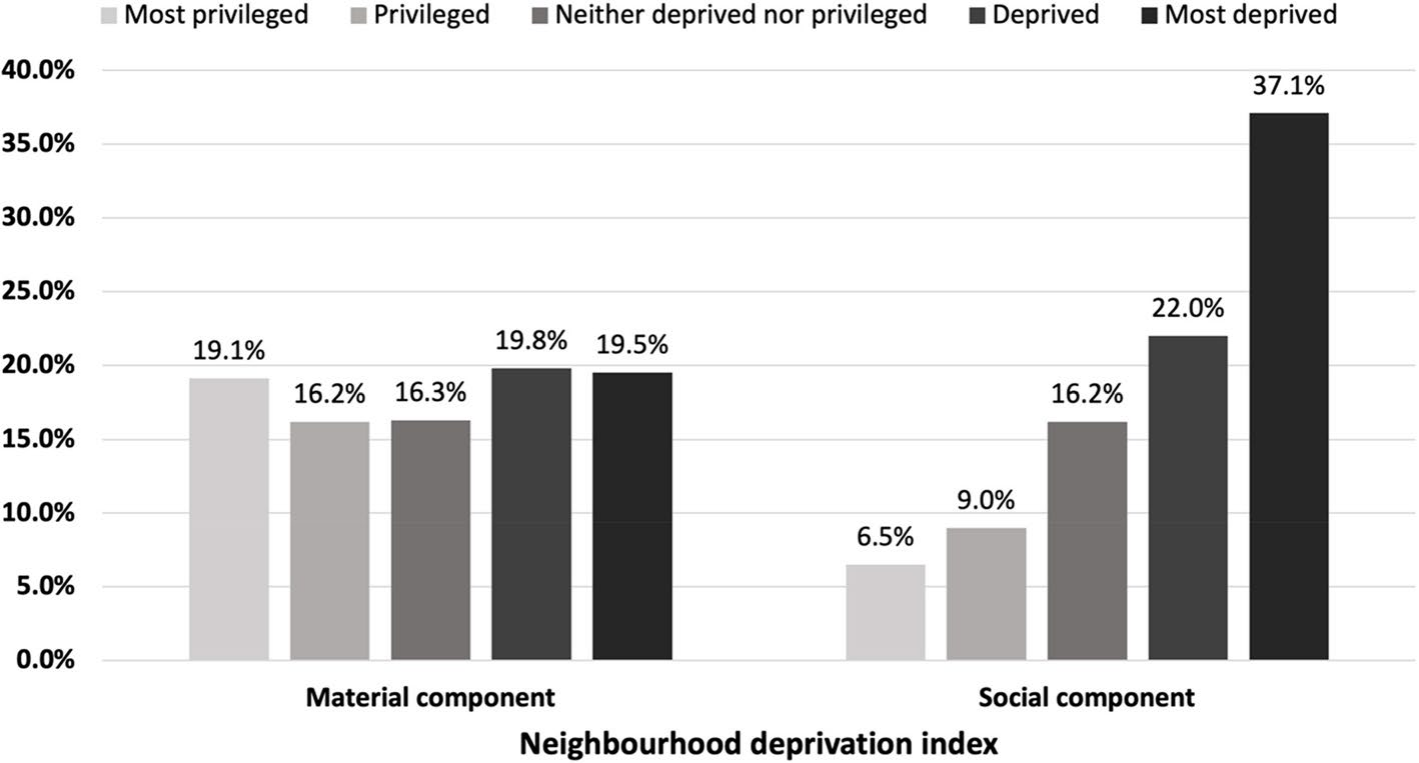
Overall distribution of retail cannabis stores by neighbourhood deprivation across Canada in September 2021 (*n* = 2477)*. *Stores with unassigned neighbourhood deprivation not included in figure (*n *= 226, 9.1%)

At the national level, there were approximately 8.0 retail cannabis stores per 100,000 individuals age 15 + in September 2021. The distribution varied from a low of 0.8 in Quebec to a high of 19.7 in Alberta (Table 1). The distribution of retail cannabis stores was relatively evenly distributed across all levels of material deprivation, ranging from 16.1% in ‘privileged’ neighbourhoods to 19.8% in ‘deprived’ neighbourhoods (Fig. 1). However, almost 60% of stores were in neighbourhoods which were characterized as ‘most socially’ and ‘socially’ deprived, i.e. neighbourhoods having higher proportions of people living alone, divorced/widowed, or single-parent families.

**Table 1:** Distribution of legal retail cannabis stores by neighbourhood deprivation in provinces and territories across Canada-September 2021 (*n* = 2477)

**Store model type**	**Private**							**Public**					**Hybrid**
	**ON**	**AB**	**MB**	**SK**	**NL**	**YK**	**NU**	**QC**	**NS**	**NB**	**NWT**	**PE**	**BC**
**Number of stores **	1042	680	112	106	33	5	1	60	33	20	6	4	375
**Stores per 100,000 individuals 15+**	8.7	19.7	10.3	11.7	7.5	15.0	2.7	0.8	4.0	3.0	18.4	3.1	8.8
**Percentage of stores by neighbourhood deprivation**													
***Material Deprivation***	**% (n)**	**% (n)**	**% (n)**	**% (n)**	**% (n)**	**% (n)**	**% (n)**	**% (n)**	**% (n)**	**% (n)**	**% (n)**	**% (n)**	**% (n)**
Most privileged	21.1 (220)	19.1 (130)	6.3 (7)	14.2 (15)	18.2 (6)	20.0 (1)	0	21.7 (13)	15.2 (5)	20.0 (4)	16.7 (1)	0	18.9 (71)
Privileged	13.3 (139)	16.5 (112)	24.1 (27)	21.7 (23)	33.3 (11)	40.0 (2)	100 (1)	18.3 (11)	27.3 (9)	20.0 (4)	33.3 (2)	0	16.0 (60)
Not deprived or privileged	15.1 (157)	17.1 (116)	19.6 (22)	17.0 (18)	15.2 (5)	20.0 (1)	0	18.3 (11)	18.2 (6)	15.0 (3)	16.7 (1)	50.0 (2)	16.5 (62)
Deprived	20.3 (211)	19.7 (134)	23.2 (26)	21.7 (23)	15.2 (5)	0	0	13.3 (8)	12.1 (4)	35.0 (7)	0	50.0 (2)	18.9 (71)
Most deprived	22.6 (235)	14.4 (98)	18.8 (21)	17.0 (18)	15.2 (5)	20.0 (1)	0	18.3 (11)	18.2 (6)	5.0 (1)	16.7 (1)	0	22.7 (85)
***Social Deprivation***													
Most privileged	5.4 (56)	7.2 (49)	8.9 (10)	7.6 (8)	24.4 (8)	0	0	6.7 (4)	3.0 (1)	5.0 (1)	0	0	5.8 (22)
Privileged	7.9 (82)	10.9 (74)	12.5 (14)	8.5 (9)	18.2 (6)	0	100 (1)	6.7 (4)	6.1 (2)	15.0 (3)	16.7 (1)	25.0 (1)	7.2 (27)
Not deprived or privileged	15.7 (164)	16.6 (113)	15.2 (17)	16.0 (17)	21.2 (7)	0	0	21.7 (13)	12.1 (4)	15.0 (3)	50.0 (3)	0	16.3 (61)
Deprived	23.0 (240)	18.2 (124)	23.2 (26)	25.5 (27)	24.2 (8)	20.0 (1)	0	32.7 (19)	39.4 (13)	30.0 (6)	0	50.0 (2)	21.3 (80)
Most deprived	40.3 (420)	33.8 (230)	32.1 (36)	34.0 (36)	9.1 (3)	80.0 (4)	0	23.3 (13)	30.3 (10)	30.0 (6)	16.7 (1)	25.0 (1)	42.4 (159)
Deprivation score not assigned	7.7 (80)	13.2 (90)	8.0 (9)	8.5 (9)	3.0 (1)	0	0	10.0 (6)	9.1 (3)	5.0 (1)	16.7 (1)	0	6.9 (26)

The prairie provinces (Alberta, Manitoba, and Saskatchewan), as well as the Yukon and Northwest Territories, had more stores per capita than other provinces (Table 1). Among provinces with at least 20 stores, the distribution of stores across levels of material and social deprivation tended to follow the overall national patterns, with some exceptions (Table 1). For example, in Manitoba, only 6.4% of stores were located in ‘materially privileged’ neighbourhoods, substantially lower than other provinces. In terms of social deprivation, Newfoundland was the only province that did not follow the national trend; specifically, fewer stores were located in the ‘most socially deprived’ neighbourhoods (9.1%), and a higher proportion of stores were in the ‘most socially privileged’ neighbourhoods (24.4%).

In two of the three largest census metropolitan areas in Canada, Toronto and Vancouver, more stores were located in the ‘most materially privileged’ neighbourhoods and in the ‘most socially deprived’ and ‘socially deprived’ neighbourhoods (Table 2). In Montreal, there were fewer stores in the ‘most materially deprived’ neighbourhoods; however, there were more stores in the ‘most socially deprived’ neighbourhoods.

**Table 2:** Distribution of legal retail cannabis stores by neighbourhood deprivation in the three largest census metropolitan areas^a^ in Canada - September 2021 (*n* = 442)

**Store model type**	**Private**	**Public**	**Hybrid**
	**Toronto, Ontario**	**Montreal, Quebec**	**Vancouver, British Columbia**
**Number of stores **	352	22	68
**Percentage of stores by neighbourhood deprivation**			
***Material Deprivation***	**% (n)**	**% (n)**	**% (n)**
Most privileged	31.3 (110)	18.2 (4)	44.1 (30)
Privileged	17.6 (62)	18.2 (4)	13.2 (9)
Not privileged or deprived	12.8 (45)	27.3 (6)	16.2 (11)
Deprived	20.7 (73)	22.7 (5)	10.3 (7)
Most deprived	16.8 (59)	9.1 (2)	16.2 (11)
***Social Deprivation***			
Most privileged	4.6 (16)	4.6 (1)	4.4 (3)
Privileged	5.7 (20)	18.2 (4)	5.9 (4)
Not privileged or deprived	16.2 (57)	18.2 (4)	20.6 (14)
Deprived	35.5 (125)	13.6 (3)	33.9 (23)
Most deprived	37.2 (131)	40.9 (9)	35.3 (24)
Deprivation score not assigned	0.9 (1)	4.6 (1)	0

^a^Census metropolitan areas are based on 2016 census data

## Discussion

Overall, the number of cannabis stores in Canada more than doubled over a 12-month period, from 3.7 per 100,000 individuals age 15 + in October 2020 (Myran et al. 2022) to 8.0 per 100,00 individuals 15 + in September 2021. In general, there were more stores per capita in provinces with a private or hybrid retail model than a public model.

When looking at the distribution of stores based on material and social deprivation, two trends emerged. Since the distribution of stores is based on quintiles for the dissemination areas, equitable distribution across deprivation levels would be equivalent to 20% of stores within each quintile. The pattern for material deprivation was very close to this, with a range of 16–20% within each of the five levels of deprivation. Data from Toronto and Vancouver, two of the three largest census metropolitan areas, revealed that in these areas more stores were located in the most materially privileged neighbourhoods. This is in contrast to previous work in Canada which reported that by October 2020 retail density of stores was greater in areas around low-income neighbourhoods (Myran et al. 2022). As the current study did not assess density of stores, but rather distribution of stores across levels of material deprivation, methodological differences may account for the discrepant findings. In addition, given that the INSPQ measure of deprivation uses several factors to establish material deprivation, rather than income alone, this may in part account for the differences noted. Also, as we used retail data up to and including September 2021, the patterns of distribution may have changed during this period of substantial growth in retail availability.

The distribution of legal cannabis stores based on material deprivation of neighbourhoods in Canada contrasts with findings from legal markets in US states. Several studies have found that both medical cannabis dispensaries and recreational cannabis outlets were more likely to be located in low-income neighbourhoods (Mair et al. 2015; Amiri et al. 2019). It has been hypothesized that this may be attributable to zoning restrictions, demand for cannabis, and co-location with alcohol outlets as well differences in the availability to resources to deter the establishment of stores (Firth et al. 2020). In Canada, zoning regulations in most provinces allow for brick-and-mortar stores to be located where any other retail outlet could be located, provided some jurisdictional guidelines are followed. These regulations may result in the more equitable distribution across neighbourhoods which is driven by factors such as market demand, visibility, and consumer convenience.

Approximately 60% of cannabis stores were located within socially deprived neighbourhoods, which are characterized by more people living alone. Although there are no clear data available on how retailers have decided where to establish a store, it is not surprising that more stores would be found in areas with more people living alone, particularly as these areas tend to have younger adults with higher levels of educational attainment and employment, as well as more individuals living in high density housing (Tang et al. 2019). Large urban areas, such as Toronto, tend to have a greater proportion of people living alone than the national average, as well as higher levels of retail density (City of Toronto 2017; Draaisma 2021). The higher proportion of cannabis stores in these areas may be explained by higher rates of cannabis use among young people and higher levels of demand for cannabis stores in large urban centres. In addition, urban neighbourhoods may have more areas zoned for mixed commercial and residential land use. As stores would not be located in dissemination areas which are zoned for residential land use, and where couples and families with and without children may be more likely to reside, it is reasonable to find that there are fewer stores in more ‘socially privileged’ areas. Future research should examine whether there is higher demand for legal cannabis in more socially deprived neighbourhoods as well as the characteristics of these neighbourhoods, including the role of urbanization and population density. In addition, research should consider if those who reside in neighbourhoods which are more socially deprived are differentially impacted in terms of cannabis use and other potential harmful outcomes associated with exposure to cannabis retail stores. As one of the primary goals of legalization is to reduce the illicit market, ensuring that legal cannabis is available where demand is high must be considered against potential risks.

### Limitations

The current study has several limitations. First, approximately 9% of stores were not classified by neighbourhood deprivation because of unmatched postal codes. Postal codes for non-residential areas, those in dissemination areas with smaller populations where no census income data is available, and new postal codes in areas which were developed after 2016 and thus not included in the 2016 census, are possible reasons for unmatched data. Second, the area delineated by dissemination areas may not precisely represent the area residents consider their ‘neighbourhood’ and defining ‘neighbourhood’ differently could result in different deprivation scores. This may be particularly relevant in rural areas where population density is less. Third, the measure of social deprivation likely reflects living situation rather than capturing elements of social support and community cohesion. Nonetheless, the social deprivation index does draw attention to factors outside of material deprivation which should be considered when assessing issues related to equity (Ross et al. 2013). Future research with individuals should include measures to assess social support and community connection when considering the impact of social deprivation on cannabis use outcomes. Fourth, the current work did not consider the availability of legal cannabis through online platforms. While this likely impacts accessibility, the focus of this study was to consider the distribution of physical stores and the potential impact this may have within neighbourhoods. Lastly, the current study did not examine the distribution of unlicensed cannabis outlets, which also has the potential to impact outcomes, particularly for those in more deprived neighbourhoods (Pedersen et al. 2021).

## Conclusion

The current study findings that legal retail cannabis outlets were relatively evenly distributed across neighbourhoods at all levels of material deprivation and that more stores were in socially deprived neighbourhoods were both unexpected. As the legal cannabis market in Canada continues to grow it will be important to continue to monitor the distribution of legal cannabis stores to determine if the patterns remain the same or change over time. This is particularly true in terms of the greater distribution of stores in more socially deprived neighbourhoods as increased exposure may have implications for cannabis use. Future research should also examine the impact of cannabis stores in terms of the balance between displacing the illicit market, without promoting greater consumption.

## Data Availability

The datasets used and/or analysed during the current study are available from the corresponding author on reasonable request.

## Abbreviations

DA: Dissemination area
ICPS: International Cannabis Policy Study
INSPQ: Institut National de Santé Publique du Québec
